# 2-Acetyl­pyridinium bromanilate

**DOI:** 10.1107/S1600536809016456

**Published:** 2009-05-07

**Authors:** Lynne H. Thomas, Bryan Boyle, Lesley A. Clive, Anna Collins, Lynsey D. Currie, Malgorzata Gogol, Claire Hastings, Andrew O. F. Jones, Jennifer L. Kennedy, Graham B. Kerr, Alastair Kidd, Lorreta M. Lawton, Susan J. Macintyre, Niall M. MacLean, Alan R. G. Martin, Kate McGonagle, Samantha Melrose, Gaius A. Rew, Colin W. Robinson, Marc Schmidtmann, Felicity B. Turnbull, Lewis G. Williams, Alan Y. Wiseman, Malgorzata H. Wocial, Chick C. Wilson

**Affiliations:** a WestCHEM, Department of Chemistry, University of Glasgow, University Avenue, Glasgow G12 8QQ, Scotland; b Department of Chemistry, University of Glasgow, University Avenue, Glasgow G12 8QQ, Scotland

## Abstract

In the crystal of the title mol­ecular salt (systematic name: 2-acetyl­pyridinium 2,5-dibromo-4-hydr­oxy-3,6-dioxocyclo­hexa-1,4-dienolate), C_7_H_8_NO^+^·C_6_HBr_2_O_4_^−^, centrosymmetric rings consisting of two cations and two anions are formed, with the components linked by alternating O—H⋯O and N—H⋯O hydrogen bonds. Short O⋯Br contacts [3.243 (2) and 3.359 (2) Å] may help to consolidate the packing.

## Related literature

For the structure of bromanilic acid, see: Robl (1987 ▶). For related structures, see: Tomura & Yamashita (2000 ▶); Zaman *et al.* (2001 ▶, 2004 ▶); Horiuchi *et al.* (2005 ▶).
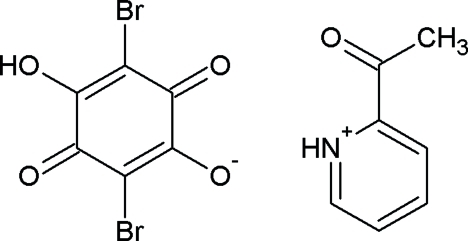


## Experimental

### 

#### Crystal data

C_7_H_8_NO^+^·C_6_HBr_2_O_4_^−^
*M* *_r_* = 419.03
Monoclinic, 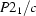

*a* = 9.1323 (5) Å
*b* = 13.3821 (7) Å
*c* = 12.2287 (7) Å
β = 112.396 (2)°
*V* = 1381.74 (13) Å^3^
*Z* = 4
Mo *K*α radiation
μ = 5.89 mm^−1^
*T* = 100 K
0.25 × 0.2 × 0.1 mm

#### Data collection

Rigaku R-AXIS RAPID IP diffractometer
Absorption correction: empirical (using intensity measurements) (*CrystalClear*; Rigaku/MSC, 2008 ▶) *T*_min_ = 0.561, *T*_max_ = 1.000 (expected range = 0.311–0.555)
17193 measured reflections
3156 independent reflections
2793 reflections with *I* > 2σ(*I*)
*R*_int_ = 0.036

#### Refinement

*R*[*F* ^2^ > 2σ(*F* ^2^)] = 0.022
*wR*(*F* ^2^) = 0.050
*S* = 1.04
3156 reflections
219 parameters
H atoms treated by a mixture of independent and constrained refinement
Δρ_max_ = 0.43 e Å^−3^
Δρ_min_ = −0.31 e Å^−3^

### 

Data collection: *CrystalClear* (Rigaku/MSC, 2008 ▶); cell refinement: *CrystalClear*; data reduction: *CrystalClear*; program(s) used to solve structure: *SHELXS97* (Sheldrick, 2008 ▶); program(s) used to refine structure: *SHELXL97* (Sheldrick, 2008 ▶); molecular graphics: *ORTEP-3* (Farrugia, 1997 ▶) and *Mercury* (Macrae *et al.*, 2006 ▶); software used to prepare material for publication: *WinGX* (Farrugia, 1999 ▶).

## Supplementary Material

Crystal structure: contains datablocks I, global. DOI: 10.1107/S1600536809016456/hb2948sup1.cif

Structure factors: contains datablocks I. DOI: 10.1107/S1600536809016456/hb2948Isup2.hkl

Additional supplementary materials:  crystallographic information; 3D view; checkCIF report

## Figures and Tables

**Table 1:** Hydrogen-bond geometry (Å, °)

*D*—H⋯*A*	*D*—H	H⋯*A*	*D*⋯*A*	*D*—H⋯*A*
O4—H1⋯O5	0.78 (3)	2.20 (3)	2.798 (2)	134 (3)
N1—H6⋯O2^i^	0.91 (3)	1.83 (3)	2.673 (2)	154 (3)

Symmetry code: (i) 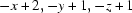.

**Fig. 1.**
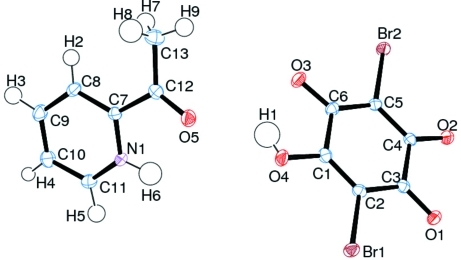
The molecular structure of (I) with displacement ellipsoids drawn at the 50% probability level.

**Fig. 2.**
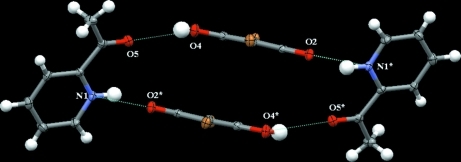
The hydrogen bonded ring between alternating bromanilic acid and acetylpyridine molecules. The hydrogen bonds are indicated by dashed lines. The * indicates the atoms are related by the symmetry code 2 - x, 1 - y, 1 - z.

**Fig. 3.**
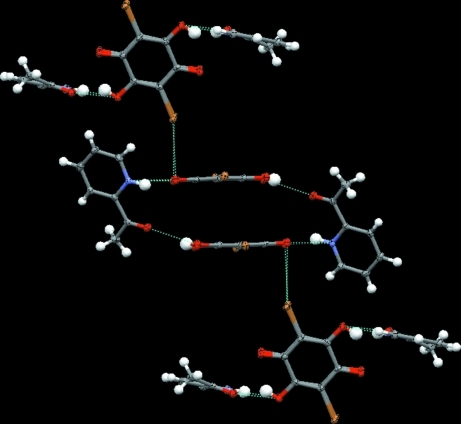
The short bromine-oxygen close contacts connecting the hydrogen bonded rings. The short contacts and hydrogen bonds are indicated by dashed lines.

## supplementary crystallographic information

### Comment

The stucture of the molecular proton-transfer salt of bromanilic acid with 2-acetylpyridine at 100 K is reported (Fig. 1). A proton is transferred from the bromanilic acid molecule to the N atom on the acetylpyridine (Fig. 1). All previously reported structures containing bromanilic acid have shown the tendency for extended chains of molecules to form. In this case, hydrogen-bonded rings are formed between alternating cations and anions (Fig. 2) and these rings are held together to form a three-dimensional structure by one Br···O close contact of 3.243 (2)Å (cf the sum of the van der Waals radii for Br and O of 3.37Å) and one on the limit of the sum of the van der Waals radii of of 3.359 (2)Å (Fig. 3). The deprotonated hydroxyl group on the bromanilic acid molecule is stabilized by forming a moderate hydrogen bond [2.673 (2)Å] with the N atom on the 2-acetylpyridine molecule to which the proton has been transferred, and a short O···Br contact with another bromanilic acid molecule. The C—O bond length to the deprotonated oxygen is notably shortened compared to that to the protonated hydroxyl group [1.253 (2)Å *versus* 1.322 (2)Å]. The longer of the two O···Br close contacts is to the C═O group on the bromanilic acid [C═O bond length 1.221 (2)Å].

### Experimental

Red blocks of (I) were grown by slow evaporation of solvent from a 1:1 solution of bromanilic acid and 2-acetylpyridine in methanol.

### Refinement

The H atoms were identified in the difference map, and their positions were freely refined. The O- and N-bonded species were allowed to refine isotropically and the C-bonded H atoms were constrained, with U_iso_(H) = 1.2U_eq_(C).

### Crystal data

**Table:**

C_7_H_8_NO^+^·C_6_HBr_2_O_4_^−^	*F*(000) = 816
*M**_r_* = 419.03	*D*_x_ = 2.014 Mg m^−^^3^
Monoclinic, *P*2_1_/*c*	Mo *K*α radiation, λ = 0.71073 Å
Hall symbol: -P 2ybc	Cell parameters from 13698 reflections
*a* = 9.1323 (5) Å	θ = 6.1–55.2°
*b* = 13.3821 (7) Å	µ = 5.89 mm^−^^1^
*c* = 12.2287 (7) Å	*T* = 100 K
β = 112.396 (2)°	Block, red
*V* = 1381.74 (13) Å^3^	0.25 × 0.2 × 0.1 mm
*Z* = 4	

### Data collection

**Table:**

Rigaku R-AXIS RAPID IP diffractometer	2793 reflections with *I* > 2σ(*I*)
graphite	*R*_int_ = 0.036
ω scans	θ_max_ = 27.5°, θ_min_ = 3.0°
Absorption correction: empirical (using intensity measurements) (*CrystalClear*; Rigaku/MSC, 2008)	*h* = −11→11
*T*_min_ = 0.561, *T*_max_ = 1.000	*k* = −17→17
17193 measured reflections	*l* = −15→15
3156 independent reflections	

### Refinement

**Table:**

Refinement on *F*^2^	Primary atom site location: structure-invariant direct methods
Least-squares matrix: full	Secondary atom site location: difference Fourier map
*R*[*F*^2^ > 2σ(*F*^2^)] = 0.022	Hydrogen site location: difference Fourier map
*wR*(*F*^2^) = 0.050	H atoms treated by a mixture of independent and constrained refinement
*S* = 1.04	*w* = 1/[σ^2^(*F*_o_^2^) + (0.0229*P*)^2^ + 0.7894*P*] where *P* = (*F*_o_^2^ + 2*F*_c_^2^)/3
3156 reflections	(Δ/σ)_max_ = 0.001
219 parameters	Δρ_max_ = 0.43 e Å^−^^3^
0 restraints	Δρ_min_ = −0.31 e Å^−^^3^

### Special details

**Table:**

Geometry. All s.u.'s (except the s.u. in the dihedral angle between two l.s. planes) are estimated using the full covariance matrix. The cell s.u.'s are taken into account individually in the estimation of s.u.'s in distances, angles and torsion angles; correlations between s.u.'s in cell parameters are only used when they are defined by crystal symmetry. An approximate (isotropic) treatment of cell s.u.'s is used for estimating s.u.'s involving l.s. planes.
Refinement. Refinement of *F*^2^ against ALL reflections. The weighted *R*-factor *wR* and goodness of fit *S* are based on *F*^2^, conventional *R*-factors *R* are based on *F*, with *F* set to zero for negative *F*^2^. The threshold expression of *F*^2^ > 2σ(*F*^2^) is used only for calculating *R*-factors(gt) *etc*. and is not relevant to the choice of reflections for refinement. *R*-factors based on *F*^2^ are statistically about twice as large as those based on *F*, and *R*- factors based on ALL data will be even larger. The isotropic displacement parameters for the hydrogen atoms involved in hydrogenbonds are refined freely. All other hydrogen atoms are refined against the atoms to which they are bonded.

### Fractional atomic coordinates and isotropic or equivalent isotropic displacement parameters (Å^2^)

**Table:**

	*x*	*y*	*z*	*U*_iso_*/*U*_eq_	
H6	0.611 (3)	0.307 (2)	0.195 (3)	0.041 (8)*	
O5	0.50649 (17)	0.44777 (11)	0.25644 (13)	0.0201 (3)	
N1	0.5396 (2)	0.29814 (12)	0.11977 (16)	0.0152 (3)	
C12	0.4282 (2)	0.45501 (15)	0.15172 (18)	0.0160 (4)	
C11	0.5549 (2)	0.22016 (16)	0.05849 (19)	0.0200 (4)	
H5	0.637 (3)	0.1735 (18)	0.102 (2)	0.024*	
C8	0.3294 (2)	0.36207 (16)	−0.04572 (18)	0.0173 (4)	
H2	0.263 (3)	0.4122 (18)	−0.076 (2)	0.021*	
C10	0.4555 (3)	0.20854 (17)	−0.0595 (2)	0.0223 (5)	
H4	0.468 (3)	0.156 (2)	−0.099 (2)	0.027*	
C7	0.4298 (2)	0.37017 (14)	0.07151 (17)	0.0145 (4)	
C9	0.3417 (3)	0.28035 (17)	−0.11190 (19)	0.0217 (4)	
H3	0.274 (3)	0.2759 (19)	−0.195 (2)	0.026*	
C13	0.3272 (3)	0.54328 (17)	0.0970 (2)	0.0232 (5)	
H9	0.351 (3)	0.595 (2)	0.152 (2)	0.028*	
H7	0.340 (3)	0.5618 (19)	0.027 (2)	0.028*	
H8	0.222 (3)	0.5245 (19)	0.076 (2)	0.028*	
H1	0.663 (4)	0.511 (2)	0.426 (3)	0.050 (10)*	
Br1	0.92699 (2)	0.416206 (15)	0.738904 (17)	0.01764 (6)	
Br2	0.98903 (2)	0.811177 (15)	0.427719 (17)	0.01869 (6)	
O1	1.19471 (16)	0.56928 (11)	0.79086 (12)	0.0180 (3)	
O2	1.22536 (16)	0.73058 (10)	0.67034 (12)	0.0186 (3)	
O3	0.73311 (16)	0.64428 (11)	0.37219 (12)	0.0198 (3)	
C5	0.9790 (2)	0.69726 (14)	0.51628 (17)	0.0150 (4)	
O4	0.70848 (17)	0.48853 (11)	0.48976 (14)	0.0197 (3)	
C1	0.8359 (2)	0.54445 (15)	0.54043 (18)	0.0152 (4)	
C6	0.8462 (2)	0.63450 (15)	0.46801 (17)	0.0149 (4)	
C4	1.1029 (2)	0.67882 (14)	0.62391 (18)	0.0139 (4)	
C3	1.0879 (2)	0.58650 (14)	0.69581 (17)	0.0143 (4)	
C2	0.9474 (2)	0.52459 (14)	0.64757 (17)	0.0145 (4)	

### Atomic displacement parameters (Å^2^)

**Table:**

	*U*^11^	*U*^22^	*U*^33^	*U*^12^	*U*^13^	*U*^23^
O5	0.0225 (8)	0.0205 (7)	0.0161 (7)	−0.0024 (6)	0.0059 (6)	−0.0021 (6)
N1	0.0126 (8)	0.0165 (8)	0.0135 (8)	0.0001 (6)	0.0014 (7)	0.0008 (7)
C12	0.0157 (10)	0.0161 (10)	0.0184 (10)	−0.0023 (7)	0.0088 (8)	0.0005 (8)
C11	0.0178 (10)	0.0179 (10)	0.0219 (11)	0.0044 (8)	0.0048 (9)	0.0002 (8)
C8	0.0129 (10)	0.0206 (10)	0.0158 (10)	0.0007 (8)	0.0024 (8)	0.0035 (8)
C10	0.0277 (12)	0.0212 (11)	0.0185 (11)	0.0005 (9)	0.0093 (9)	−0.0056 (9)
C7	0.0138 (9)	0.0143 (9)	0.0158 (10)	−0.0004 (7)	0.0062 (8)	0.0011 (8)
C9	0.0242 (11)	0.0246 (11)	0.0137 (10)	−0.0027 (9)	0.0045 (9)	−0.0002 (8)
C13	0.0271 (12)	0.0209 (11)	0.0249 (12)	0.0057 (9)	0.0135 (10)	0.0023 (9)
Br1	0.01649 (11)	0.01824 (11)	0.01617 (11)	−0.00241 (7)	0.00395 (8)	0.00493 (7)
Br2	0.02145 (12)	0.01616 (11)	0.01540 (11)	−0.00364 (7)	0.00360 (9)	0.00339 (7)
O1	0.0164 (7)	0.0188 (7)	0.0144 (7)	−0.0007 (5)	0.0011 (6)	0.0018 (6)
O2	0.0172 (7)	0.0158 (7)	0.0185 (7)	−0.0026 (5)	0.0020 (6)	0.0016 (6)
O3	0.0182 (7)	0.0229 (8)	0.0137 (7)	−0.0020 (6)	0.0008 (6)	0.0032 (6)
C5	0.0184 (10)	0.0128 (9)	0.0129 (9)	−0.0001 (7)	0.0051 (8)	0.0021 (7)
O4	0.0168 (8)	0.0208 (8)	0.0154 (7)	−0.0058 (6)	−0.0008 (6)	0.0026 (6)
C1	0.0155 (10)	0.0153 (9)	0.0154 (10)	−0.0005 (7)	0.0065 (8)	−0.0014 (8)
C6	0.0156 (10)	0.0165 (10)	0.0127 (9)	0.0016 (7)	0.0057 (8)	0.0005 (8)
C4	0.0147 (10)	0.0122 (9)	0.0152 (10)	0.0003 (7)	0.0061 (8)	−0.0013 (7)
C3	0.0164 (10)	0.0136 (9)	0.0145 (10)	0.0013 (7)	0.0077 (8)	−0.0015 (7)
C2	0.0167 (10)	0.0130 (9)	0.0151 (9)	−0.0001 (7)	0.0075 (8)	0.0012 (7)

### Geometric parameters (Å, °)

**Table:**

O5—C12	1.209 (2)	C13—H7	0.94 (3)
N1—C11	1.323 (3)	C13—H8	0.93 (3)
N1—C7	1.353 (2)	Br1—C2	1.8826 (19)
N1—H6	0.91 (3)	Br2—C5	1.8922 (19)
C12—C13	1.491 (3)	O1—C3	1.221 (2)
C12—C7	1.504 (3)	O2—C4	1.253 (2)
C11—C10	1.390 (3)	O3—C6	1.239 (2)
C11—H5	0.96 (3)	C5—C4	1.392 (3)
C8—C7	1.381 (3)	C5—C6	1.407 (3)
C8—C9	1.390 (3)	O4—C1	1.322 (2)
C8—H2	0.89 (2)	O4—H1	0.79 (3)
C10—C9	1.381 (3)	C1—C2	1.344 (3)
C10—H4	0.88 (3)	C1—C6	1.520 (3)
C9—H3	0.97 (3)	C4—C3	1.552 (3)
C13—H9	0.93 (3)	C3—C2	1.451 (3)
			
C11—N1—C7	122.38 (18)	H9—C13—H7	112 (2)
C11—N1—H6	119.2 (18)	C12—C13—H8	107.8 (16)
C7—N1—H6	118.2 (18)	H9—C13—H8	110 (2)
O5—C12—C13	123.61 (19)	H7—C13—H8	107 (2)
O5—C12—C7	118.77 (18)	C4—C5—C6	123.42 (18)
C13—C12—C7	117.62 (18)	C4—C5—Br2	119.00 (14)
N1—C11—C10	120.53 (19)	C6—C5—Br2	117.57 (14)
N1—C11—H5	115.3 (15)	C1—O4—H1	107 (2)
C10—C11—H5	124.2 (15)	O4—C1—C2	123.31 (19)
C7—C8—C9	119.83 (19)	O4—C1—C6	114.51 (17)
C7—C8—H2	117.2 (16)	C2—C1—C6	122.18 (17)
C9—C8—H2	122.9 (16)	O3—C6—C5	127.49 (19)
C9—C10—C11	118.8 (2)	O3—C6—C1	114.86 (17)
C9—C10—H4	122.0 (16)	C5—C6—C1	117.65 (17)
C11—C10—H4	119.1 (16)	O2—C4—C5	126.44 (18)
N1—C7—C8	119.03 (18)	O2—C4—C3	116.07 (17)
N1—C7—C12	116.30 (17)	C5—C4—C3	117.48 (17)
C8—C7—C12	124.67 (18)	O1—C3—C2	122.78 (18)
C10—C9—C8	119.39 (19)	O1—C3—C4	118.58 (17)
C10—C9—H3	120.6 (15)	C2—C3—C4	118.64 (17)
C8—C9—H3	119.9 (15)	C1—C2—C3	120.51 (18)
C12—C13—H9	109.4 (16)	C1—C2—Br1	121.41 (15)
C12—C13—H7	110.4 (16)	C3—C2—Br1	118.08 (14)

### Hydrogen-bond geometry (Å, °)

**Table:**

*D*—H···*A*	*D*—H	H···*A*	*D*···*A*	*D*—H···*A*
O4—H1···O5	0.78 (3)	2.20 (3)	2.798 (2)	134 (3)
N1—H6···O2^i^	0.91 (3)	1.83 (3)	2.673 (2)	154 (3)

Symmetry codes: (i) −*x*+2, −*y*+1, −*z*+1.

## References

[bb1] Allen, F. H., Kennard, O., Watson, D. G., Brammer, L., Orpen, A. G. & Taylor, R. (1995). *International Tables for Crystallography*, Vol. C, edited by A. J. C. Wilson, pp. 685–706. Dordrecht: Kluwer Academic Publishers.

[bb2] Farrugia, L. J. (1997). *J. Appl. Cryst.* **30**, 565.

[bb3] Farrugia, L. J. (1999). *J. Appl. Cryst.* **32**, 837–838.

[bb4] Horiuchi, S., Kumai, R. & Tokura, Y. (2005). *J. Am. Chem. Soc.* **127**, 5010–5011. 10.1021/ja042212s 15810822

[bb5] Macrae, C. F., Edgington, P. R., McCabe, P., Pidcock, E., Shields, G. P., Taylor, R., Towler, M. & van de Streek, J. (2006). *J. Appl. Cryst.* **39**, 453–457.

[bb6] Rigaku/MSC (2008). *CrystalClear* Rigaku/MSC, The Woodlands, Texas, USA.

[bb7] Robl, C. (1987). *Z. Kristallogr.* **180**, 249–253.

[bb8] Sheldrick, G. M. (2008). *Acta Cryst.* A**64**, 112–122. 10.1107/S0108767307043930 18156677

[bb9] Tomura, M. & Yamashita, Y. (2000). *CrystEngComm*, **2**, 92–95.

[bb10] Zaman, Md. B., Tomura, M. & Yamashita, Y. (2001). *J. Org. Chem.* **66**, 5987–5995. 10.1021/jo001746i 11529722

[bb11] Zaman, Md. B., Udachin, K. A. & Ripmeester, J. A. (2004). *Cryst. Growth Des.* **4**, 585–589.

